# Photobiomodulation Can Enhance Stem Cell Viability in Cochlea with Auditory Neuropathy but Does Not Restore Hearing

**DOI:** 10.1155/2023/6845571

**Published:** 2023-11-15

**Authors:** So-Young Chang, Eunjeong Kim, Nathaniel T. Carpena, Jae-Hun Lee, Doo Hee Kim, Min Young Lee

**Affiliations:** ^1^Beckman Laser Institute Korea, Dankook University, Cheonan 31116, Republic of Korea; ^2^Department of Biological Science, College of Science & Technology, Dankook University, Cheonan 31116, Republic of Korea; ^3^Department of Otolaryngology-Head & Neck Surgery, College of Medicine, Dankook University, Cheonan 31116, Republic of Korea; ^4^Center for Cognition and Sociality, Institute for Basic Science (IBS), Daejeon, Republic of Korea; ^5^TODOC Co. Ltd., Seoul, Republic of Korea

## Abstract

Sensorineural hearing loss is very difficult to treat. Currently, one of the techniques used for hearing rehabilitation is a cochlear implant that can transform sound into electrical signals instead of inner ear hair cells. However, the prognosis remains very poor if sufficient auditory nerve cells are not secured. In this study, the effect of mouse embryonic stem cells (mESC) and photobiomodulation (PBM) combined treatment on auditory function and auditory nerve cells in a secondary neuropathy animal model was investigated. To confirm the engraftment of stem cells in vitro, cochlear explants were treated with kanamycin (KM) to mimic nerve damage and then cocultured with GFP-mESC. GFP-mESCs were observed to have attached and integrated into the explanted samples. An animal model for secondary neurodegeneration was achieved by KM treatment and was treated by a combination therapy of GFP-mESC and NIR-PBM at 8 weeks of KM treatment. Hearing recovery by functional testing using auditory brain stem response (ABR) and eABR was measured as well as morphological changes and epifluorescence analysis were conducted after 2 weeks of combination therapy. KM treatment elevated the hearing threshold at 70–80 dB and even after the combination treatment with GFP-mESC and PBM was applied, the auditory function was not restored. In addition, the stem cells transplanted into cochlea has exponentially increased due to PBM treatment although did not produce any malignancy. This study confirmed that the combined treatment with mESC and PBM could not improve hearing or increase the response of the auditory nerve. Nevertheless, it is noteworthy in this study that the cells are distributed in most cochlear tissues and the proliferation of stem cells was very active in animals irradiated with PBM compared to other groups wherein the stem cells had disappeared immediately after transplantation or existed for only a short period of time.

## 1. Introduction

Social isolation due to hearing loss can lead to cognitive impairment, especially for the aging population [1–3]. Kanamycin is one of the antibiotics used to treat bacterial infections. Inappropriate use of kanamycin is known to cause side effects such as hearing loss or balance disorders. No clear clinical treatment is available for sensorineural hearing loss, which causes permanent intractable disease [4–6]. However, a described cochlear implantation technique that provides transmission of electrical signals to the spiral nerve nucleus, instead of to damaged hair cells, has been used successfully in patients with irreversible hearing loss [7–9].

To achieve positive hearing outcomes after cochlear implantation, rehabilitation to maintain and regenerate the nerve cells of the spiral ganglion are very important [10–13]. Nerve regeneration using pluripotent stem cells (SCs) can be used to treat hearing loss [2, 14–16]. In the auditory area, successful SC transplantation that targets the auditory organ has been reported wherein the SCs transplanted into the cochlea migrated to the spiral nerve nucleus [17, 18]. In our previous study, we confirmed the survival and distribution of mouse embryonic stem cells (mESCs) that we transplanted into the cochlea through the scala tympani (ST) in an animal model of chronic auditory nerve deafness caused by acute auditory and secondary degenerative nerve damage [19]. However, this SC treatment has two limitations. The first is related to the decrease in ability of the SCs to survive and differentiate; SCs transplanted from a donor to a host may be recognized as foreign substances and rejected or may not differentiate properly into the desired target cells. The second limitation is related to tumorigenicity; transplanted SCs that do not differentiate and maintain only by proliferation may compromise the structure and function of the original tissue, resulting in damage or even cancer [20–22]. These factors need to be addressed to improve the efficiency of SC therapy for sensorineural hearing loss.

Researchers conducting auditory studies have reported that photobiomodulation (PBM) promotes differentiation of SCs into sensory and neural cells, and that external light energy can be delivered to the inner ear [23–25]. PBM is a therapeutic approach to specific diseases based on light energy. PBM induces effects to the target tissue by applying low-level light energy which generally does not generate heat [26]. In addition, PBM is known to be an effective approach for treating pain [27], wound healing [28], neural regeneration [29], and anti-inflammation [30]. The wavelength used for the light source is within the 600–1,000 nm spectrum, and red or near-infrared (NIR) rays are mostly used. Although lasers were mainly used, light-emitting diodes (LEDs) have become popular in recent years [31, 32]. Light irradiated to tissues or cells is absorbed by chromophores such as cytochrome-c-oxidase located inside mitochondria. The absorbed energy then induces cellular biostimulation through various signaling pathways.

In addition, we indirectly demonstrated the effect of PBM on stem cell differentiation using inner ear organoids and transcriptome analysis [33]. Other published papers have also already demonstrated the proneural effects of photobiomodulation on hippocampal neurogenesis [34] and synaptic formation [35]. Therefore, it is necessary to confirm whether the fusion in application of stem cell and PBM can effectively regenerate spiral ganglion neurons.

In this study, we confirmed the occurrence of histological changes in the peripheral auditory organs and auditory function after the combination therapy of mouse embryonic stem cell (mESC) transplantation and PBM in an animal model of secondary nerve degeneration. Histological staining showed improvement in SC viability.

However, functional analyses showed no improvement in hearing. The explosive proliferation of transplanted SCs due to PBM was confirmed; this proliferation resulted in differentiation into cells other than auditory cells, but not malignant cells.

## 2. Materials and Methods

### 2.1. Animals

Thirty-one 5-week-old C57BL/c6 mice were used for the animal study. The animals were divided into four groups: untreated control (*n* = 10), kanamycin only (KM, *n* = 11), KM plus SCs (KM + SC, *n* = 7), and KM plus SCs plus PBM (KM + SC + PBM, *n* = 3).

**Figure 1:**
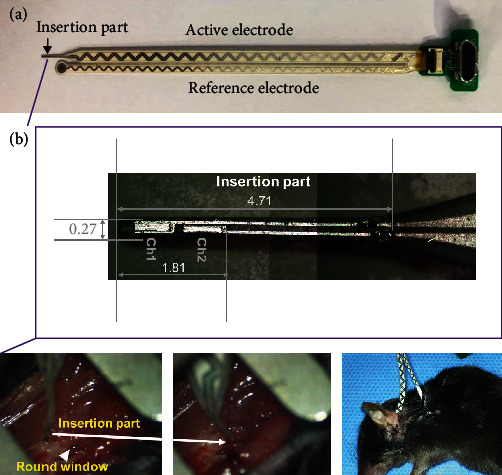
Intracochlear electrode. (a) The electroassembly. (b) Detailed view of the inserted part and electrode injection. The conduction part is made of platinum and the backbone is made of liquid polymer crystal covered with silicone elastomer. The five-micro-pin connector was assembled to deliver electrical stimulation. After exposure of the round window (RW, arrowheads) membrane, the stimulus electrode was inserted, and a reference electrode was placed in the skin near the neck for monopolar stimulation.

The ototoxic group was created by unilateral delivery of an ototoxic agent to the left ear. Before the surgery, all animals were completely anesthetized by intramuscular injection of an anesthetic solution (0.1 mL/20 g) prepared by diluting a 3 : 1 mixture of Zoletil (Virbac, Carros Cedex, France) and Rompun (Bayer, Leverkusen, Germany) in a 1 : 3 mixture of normal saline. All surgeries including mESC implantation and PBM were performed only to the left ear. Auditory function was assessed by measuring the auditory brainstem response (ABR) and auditory nerve response via electric auditory brain response (eABR) before and after the ototoxic agent injection and combination treatment with mESC transplantation and PBM (Figure 1(a)). Animal care and all experiments were performed according to the protocol approved by the Animal Care and Use Committee of Dankook University (approval number DKU-21-013).

### 2.2. Stem Cells

Green fluorescent protein (GFP)-tagged mESCs in passage 20 (donated by Professor Shim Ho-seop at Dankook University, Cheonan, Korea) were utilized for the in vitro study.

The cells were cultured and maintained using the protocol described in our previous paper [19], at 37°C in a 5% CO_2_ humidified incubator. The mESC medium consisted of high-glucose Dulbecco's modified Eagle's medium (DMEM; Gibco, Life Technologies Corporation, Grand Island, NY, USA) supplemented with 15% (v/v) heat-inactivated fetal bovine serum (FBS; ATCC, Manassas, VA, USA), 0.1 mM *β*-mercaptoethanol (Gibco, Invitrogen, Carlsbad, CA, USA), 0.1 mM GlutaMAX (Gibco), 0.1 mM ES qualified nonessential amino acid (Welgene, Daegu, Korea), 1% penicillin-streptomycin (ATCC), 1,000 U/mL leukemia inhibitory factor (Merck Millipore, Burlington, MA, USA), 0.033% CHIR99021 (Tocris Bioscience, Bristol, UK), and 0.125% PD035901 (Tocris Bioscience).

### 2.3. Cochlear Explantation and SC Coculture

Cochlear explantation was performed in postnatal days 4 and 5 Sprague–Dawley rats. The animals were sanitized with 70% ethanol and decapitated and cochleae from both ears were harvested and the bony capsule was gently removed using microforceps. The basilar membrane was dissected, and the tectorial membrane was detached. The basilar membrane including the organ of Corti (OC) was attached to a 35-mm culture dish by scratching the remaining tissue with an insulin syringe. The explants were incubated in culture medium (DMEM, 5% FBS, and 1% ampicillin) at 37°C with 5% CO_2_.

After overnight incubation, each explanted cochlea was treated with 500 mM KM (K-1377; Sigma Aldrich, St. Louis, MO, USA) for 2 hr to induce hair cell loss. One day after KM treatment, to ensure the chance of the mESCs landing on the explants within the large area of the culture plate, 1 × 10^7^ cells/mL mESCs were plated into each explant and cocultured for 3 days.

The cocultured mESCs and explanted cochlea were washed with phosphate-buffered saline (PBS), fixed with 4% paraformaldehyde (PFA) for 15 min and then incubated in blocking solution (5% normal goat serum (NGS) with 0.3% Triton-X in 0.1 M PBS) for 1 hr. After blocking, the primary antibodies and the corresponding secondary antibodies were applied.

### 2.4. Mouse Model of Auditory Neural Hearing Loss

To induce secondary neurodegeneration with KM, the posterior wall of the left ear was dissected until the bulla appeared. The bulla was carefully removed until the round window (RW) of the cochlea was exposed. Then, 5 *µ*L of a 150-mg/kg concentration of KM solution was injected into the RW for delivery to the ST using a microcannula tube connected to a Hamilton syringe. The incision was then sutured, and the mouse was allowed to rest on a warm pad until it could move normally. To evaluate hearing changes, the ABR was assessed before surgery as well as 2, 4, and 8 weeks after KM treatment and 1 and 2 weeks after combination therapy (9 and 10 weeks after KM treatment).

### 2.5. Green Fluorescent Protein-Tagged Mouse Embryonic Stem Cell Transplantation

For the mESC transplantation experiments, GFP-tagged mESC transplantation was performed 8 weeks after KM surgery using the procedure described by Chang [19]. Briefly, the posterior wall of the left ear of each anesthetized mouse was recut to confirm the RW position, and then a total of 60,000 mESCs (3 *μ*L of 2 × 10^4^ cells/*μ*L) was injected into the RW of the cochlea. After SC transplantation, the incision was sutured in the same manner as used to induce auditory neural hearing loss.

### 2.6. Photobiomodulation

PBM was performed in animals with secondary neurodegeneration after GFP-tagged mESC transplantation using a near-infrared (NIR) diode laser with a wavelength of 808 nm (Won Tech, Daejeon, South Korea). It was applied to the left ear at 40 mW/cm^2^ for 1 hr/day for 5 days. Before the animals were irradiated, the laser was calibrated and checked using a FieldMax II-To Meter (Coherent Inc., Salem, NH, USA) and a detector (Powermax; Coherent Inc.). An optical fiber (core diameter 62.5 *μ*m, cladding diameter 125 *μ*m) was inserted into the ear canal through a hollow tube until its tip was ∼1 mm from the eardrum surface.

### 2.7. Auditory Brainstem Response Measurement

Hearing changes were evaluated by measuring ABRs (RZ6 Multi-I/O Processor; Tucker Davis Technologies, Alachua, FL, USA) using tone bursts and specific stimulation frequencies. Each mouse was anesthetized and placed in a sound chamber. Three needle electrodes were inserted at the vertex (active) and under both auricles (reference and ground). Hearing thresholds were measured with tone burst stimulation at 8, 16, and 32 kHz through a tube inserted into the left ear. The ABR waveform was assembled by averaging 512 signals at 10 ms/step, measured from 80 to 10 dB SPL at intervals of 5 and 10 dB.

### 2.8. Electrical Auditory Brainstem Response Measurement

eABRs were measured using a neural stimulator (NerveOn; TODOC Co. Ltd., Seoul, Republic of Korea) and ABR measurement system (RG6; Tucker Davis Technologies). An intracochlear platinum electrode (inserted portion diameter 0.27 mm; TODOC Co. Ltd.) was used to stimulate the spiral ganglion neurons. Impedance values (mean ± standard deviation) for channels 1, 2, and the reference electrodes were 6.12 ± 1.53, 5.33 ± 1.69, and 0.48 ± 0.19 kΩ, respectively. To measure the eABR, the animals were anesthetized, and a postauricular incision was made in the left ear. The temporal bone was drilled with blunt forceps and the cochlea was exposed. The RW membrane was punctured with a needle, and the stimulus electrode (TODOC Co. Ltd.) was inserted (Figure 1). The reference electrode was placed in the skin near the neck for monopolar stimulation. During measurement, the bulla was covered with incised skin.

Biphasic electrical stimulation pulses (rate 20 Hz, width/phase 100 *µ*s) were generated and transmitted to the electrode from 0 to 1,000 *μ*V in 200-*μ*V steps. eABRs were recorded (with 30–3,000-Hz bandpass filtering) and interpreted using BiosigRZ software (Tucker Davis Technologies). Responses with 500 sweeps were represented using a 10-ms-window scale. The response threshold was set as the lowest stimulus level with an observable peak. After eABR measurement, the animals were sacrificed and the cochleas were harvested for histological analysis.

### 2.9. Histological and Epifluorescence Analyses

The cochleas were prepared for histology and epifluorescence using a method similar to that described by Chang [19]. Briefly, the cochleas were harvested from mice in each group after sacrifice, fixed in 4% PFA, and then decalcified in 0.1 M ethylenediaminetetraacetic acid (EDTA, GeneAll Biotechnology, Seoul, Korea) solution for 4 days at room temperature (RT). Next, the cochleae were washed with PBS for 3 hr to terminate decalcification, and then embedded in paraffin blocks. The blocks were sliced into 5-*μ*m-thick sections and stained with hematoxylin and eosin (H&E) or subjected to epifluorescence analysis. Histological changes in the peripheral auditory organs after mESC transplantation in the auditory neural hearing loss model were evaluated by microscopic observation (BX53; Olympus, Tokyo, Japan).

For the epifluorescence evaluation, the slides were dried for 10 min, washed three times with PBS for 10 min, and blocked for 1 hr at RT with 5% normal goat serum (NGS, S-1000, Vector Laboratories, Burlington, Canada) and 0.3% Triton X-100 (X100, Sigma–Aldrich) to prevent nonspecific binding. To investigate whether the transplanted cells in the cochlea turned into spiral ganglion neurons, the slides were incubated overnight with a neurofilament heavy chain (NFH) antibody (1 : 200; AB5539, Merck, Darmstadt, Germany) at 4°C. The following day, the slides were washed three times with PBS for 5 min and then incubated with secondary antibody (Alexa Fluor 568-conjugated goat anti-chicken IgY, 1 : 1,000; A-11041, Invitrogen, Waltham, MA, USA) for 1 hr. The nuclei were stained with 4′,6-diamidino-2-phenylindole (DAPI).

To evaluate the characteristics of GFP-positive cells after mESC transplantation and PBM, OCT4, NANOG, GFAP, and Nestin expression in the cochleas of the auditory neurodegeneration model was investigated. The slides were incubated overnight at 4°C with OCT4 (1 : 200; ab19857, Abcam, Cambridge, UK), NANOG (1 : 100; PA5-20889, Thermo Fisher Scientific, Waltham, MA, USA), (GFAP 1 : 200; ab7260, Abcam), and Nestin (1 : 200; 4D11, Novus Biologicals, USA). Then, the samples were washed three times with PBS for 10 min and incubated with a secondary antibody (Alexa Fluor 568-conjugated goat anti-rabbit IgG, 1 : 1,000; A-11011, Alexa Fluor 568-conjugated goat antimouse IgG1, 1 : 1,000; A-21124, Thermo Fisher Scientific) for 1 hr.

To investigate the teratoma formation after mESC transplantation and PBM, SOX1 expression in the cochleas of the auditory neurodegeneration model was evaluated. SOX1 (1 : 200, AF3369, R&D systems, MN, USA) and secondary antibody (1 : 1,000; A-11079, Alexa Fluor 568-conjugated rabbit anti-goat IgG, Thermo Fisher Scientific) were stained in the same manner as above.

Representative images were obtained using a confocal microscope (FV3000, Olympus Life Science, PA, USA) after mounting with Vecta Shield medium with DAPI (H-1200; Vector Laboratories, Burlingame, CA, USA).

### 2.10. Statistical Analysis

The results are expressed as means ± standard deviations and were analyzed using GraphPad Prism 8.0 (GraphPad Software, Inc., La Jolla, CA, USA) and SPSS 18.0 (IBM, Armonk, NY, USA) softwares. The Kolmogorov–Smirnov test was used to determine whether the data were parametric. Two-way analysis of variance with post hoc Bonferroni tests was performed to analyze the ABR thresholds. Two-tailed Mann–Whitney *U* tests (nonparametric) and unpaired nonparametric *t*-tests were performed to compare viable cells in OC between the KM only and KM-SC-PBM groups. To analyze epi-fluorescence (NFH, GFAP, Nestin), nonparametric one-way ANOVA was performed and Tukey's multiple comparison test was used as the post hoc test. *p* values < 0.05 were considered to be significant.

## 3. Results

### 3.1. Stem Cells Transplanted after Nerve Damage Induction Can Survive and Connect to the Host Explant

**Figure 2:**
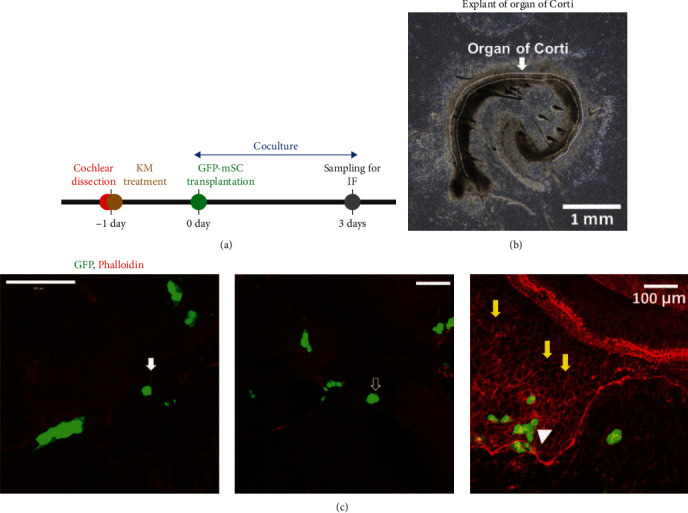
Spontaneous cell–tissue connection following stem cell administration after the induction of nerve damage. (a) Schedule for the coculture of the explants with green fluorescent protein (GFP)-tagged mouse embryonic stem cells (mESCs). The nerves of the organ of Corti (OC) explants from P4-5 mice were damaged with 500 mM kanamycin (KM) at 1 day. GFP-tagged mESCs (1 × 10^7^ cells/mL) were injected into the explants and cocultured for 3 days. (b) Representative differential interference contrast (DIC) image of a KM-treated explant after 3 days of coculture. DIC revealed damaged hair cell layers. (c) Representative epifluorescence image of a KM-treated explant after 3 days of coculture. After coculture, the samples were fixed and subjected to epifluorescence with phalloidin red. Some ESCs exhibited axon-like structures (solid white arrows) above the tissue (unfilled arrows) or between the tissue (arrowheads), suggesting integration into the explants. (Yellow arrow: flat epithelium). Scale bar = 1 mm, 100 *µ*M.

To investigate the cell characteristics following SC administration to the nerve-damaged explants with 500 mM KM, the OC explants and GFP-tagged mESCs were cocultured for 3 days after KM treatment (Figure 2(a)). Examination of the cochlear explants under differential interference contrast microscopy (Figure 2(b)) and confocal microscope revealed that the hair cell layers had been damaged by KM as demonstrated by flat epithelium (Figure 2(c)).

After 3 days of coculture of the KM-treated OC and GFP-tagged mESCs, GFP-positive cells were observed to be connected and in contact with the explants and had become integrated with the host tissue (Figure 2(c), arrowhead). In addition, some of the transplanted mESCs had spontaneously formed the cytoplasmic extension structures similar to axons (Figure 2(c), solid white arrow).

### 3.2. Combination Therapy with mESCs and PBM Did Not Improve Auditory Function in the Secondary Neurodegeneration Model

**Figure 3:**
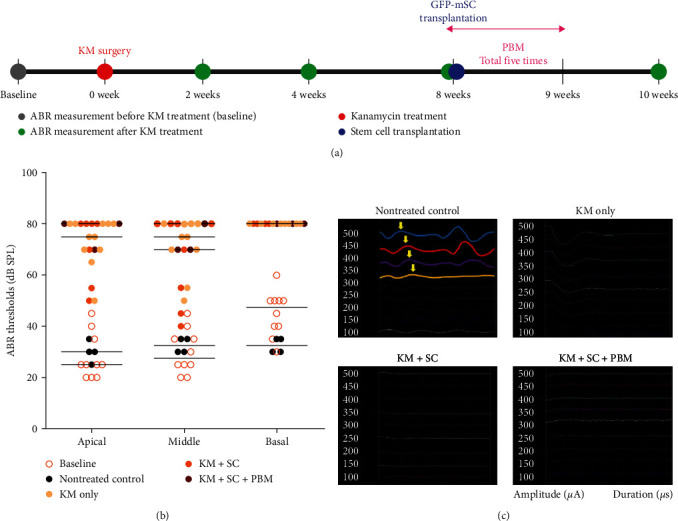
Auditory function after combination therapy with mouse embryonic stem cells (mESCs) and photobiomodulation (PBM) in a secondary neurodegeneration model. (a) Scheme for combination therapy with mESCs and PBM: Secondary neurodegeneration was induced in adult C57BL/c6 mice using kanamycin (KM). Green fluorescent protein (GFP)-tagged mESCs were transplanted into the cochlea, followed by near-infrared (NIR)-PBM irradiation of the cochlea, at 8 weeks after KM surgery. For PBM, an NIR laser with a wavelength of 808 nm was used to irradiate the cochlea through the tympanic membrane for 5 days. The mESCs were transplanted into the round window (RW) of the cochlea at 8 weeks after KM surgery, and assessment was performed at 1 and 2 weeks after the combination therapy. Hearing was evaluated by measuring auditory brainstem responses (ABRs) before and 2, 4, and 8 weeks after KM treatment and 1 and 2 weeks after combination therapy (9 and 10 weeks after KM treatment). (b) ABRs at 2 weeks after combination therapy (10 weeks after KM surgery). The ABR thresholds had increased in all groups at all frequencies (8, 16, and 32 kHz), reflecting no hearing improvement. (c) Electrical auditory brainstem responses (eABRs) at 2 weeks after combination therapy. Relative to baseline, no eABR waveform appeared, confirming the lack of nerve regeneration.

The basilar membrane is divided into areas responsible for each sound frequency, starting from the apex to the base part of cochlea. Auditory processing is performed in the apical turn for quiet sounds, the middle turn for intermediate sounds, and the basal turn for high sounds. Eight weeks after the induction of secondary neurodegeneration in adult C57BL/c6 mice using KM, a combination therapy of mESCs and NIR-PBM was administered. GFP-tagged mESCs were transplanted into the RW of the cochlea in an animal model of secondary nerve damage. Then, an 808-nm NIR laser with an energy intensity of 40 mW was used to irradiate through the tympanic membrane five times for a total of 5 days (Figure 3(a)).

To evaluate hearing, ABRs were measured before KM surgery then 2-, 4-, and 8-weeks postoperation as well as 1 and 2 weeks after combination therapy (9 and 10 weeks after KM treatment). At 2 weeks after KM surgery, the ABR thresholds in KM-treated ears had increased significantly to 70–80 dB at 8-, 16-, and 32-kHz frequencies; no further change was observed through 8 weeks (data not shown). At 2 weeks after combination therapy (10 weeks after KM surgery), the nontreated control group maintained the normal hearing over the same period, but the rest of the groups increased at all tested frequencies (Figure 3(b)). These results indicate that combination therapy with mESCs transplanted via the RW and NIR-PBM did not improve hearing in the secondary neurodegeneration model. To verify the ABR findings, eABRs were assessed at the same time point and showed that the waveforms in the KM-only, KM-SC, and KM-SC-PBM groups showed no peak relative to baseline (Figure 3(c)).

### 3.3. Combination Therapy with mESCs and PBM Altered the Peripheral Auditory System in a Secondary Neurodegeneration Model

**Figure 4:**
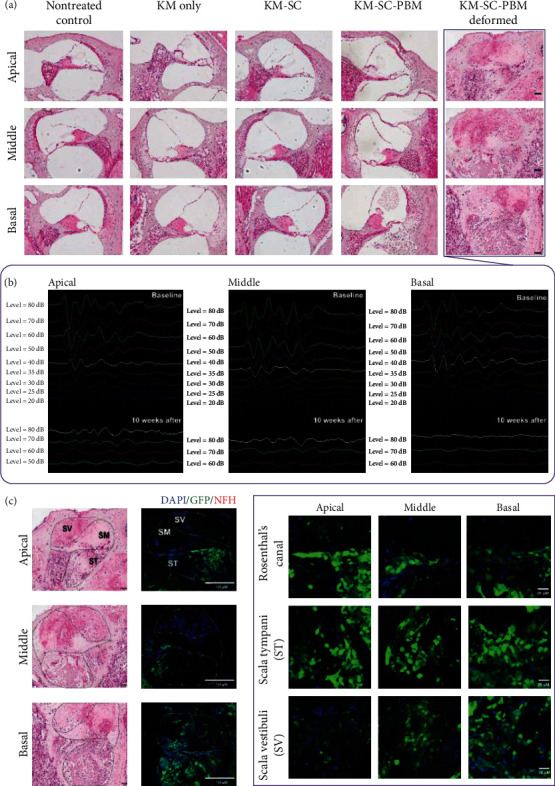
Morphological changes in the cochlea after combination therapy with GFP-mESCs and PBM in a secondary neurodegeneration model. (a) Hematoxylin and eosin (H&E) staining after 2 weeks of combination therapy (10 weeks after KM surgery). The KM group showed cell damage. More viable cells were observed in the KM-SC-PBM group after combination treatment. (b) One of the KM-SC-PBM (box) treated mice showed deformed structure in all areas of the cochlea. ABR measurement of this animal showed that hearing was still impaired after the combination therapy. (c) Deformed KM-SC-PBM showed abnormal GFP expression in all cochlear regions after 2 weeks of combination therapy. A highly magnified epifluorescence image shows GFP expression in Rosenthal's canal and in the scala media, scala tympani (ST), and scala vestibule (SV), suggesting that the SCs penetrated into various cochlear tissues. However, no neurofilament expression was observed, suggesting that the combination therapy had a biphasic effect. Scale bars = 50 *µ*M (H&E) and 100 and 20 *µ*M (IF = immunofluorescence).

Even without the restoration of hearing in mESC and PBM combination therapy of secondary neurodegeneration model, histological analyses were done to investigate whether the combination therapy had any other effects. At 2 weeks after combination therapy, the morphological changes in cochlea were observed using H&E staining and epifluorescence analysis (Figures 4(a) and 4(c)). After combination treatment, the KM alone group showed statistically significant cell damage throughout the apical, middle, and basal turns of the OC compared to the KM-SC-PBM group, and more viable cells were observed in the KM-SC-PBM group (Supplementary Figure S1).

Meanwhile, one of the KM-SC-PBM-treated mice showed deformed structure in all areas of the cochlea (Figure 4(a)). ABR measurements of deformed animals showed that hearing was also impaired after combination therapy (Figure 4(b)). In addition, the deformed KM-SC-PBM sample showed abnormal GFP expression in all cochlear regions after 2 weeks of combination therapy. A highly magnified epifluorescence image shows GFP expression in Rosenthal's canal and in the scala media, scala tympani (ST), and scala vestibule (SV), suggesting that the SCs penetrated into various cochlear tissues. However, no neurofilament expression was observed suggesting that the combination therapy had a biphasic effect (Figure 4(c)). These results suggest that the combination therapy with PBM not only improves cell survival and proliferation after transplantation but also induces abnormal cell settling and the transformation of cochlear tissue into different tissue.

**Figure 5:**
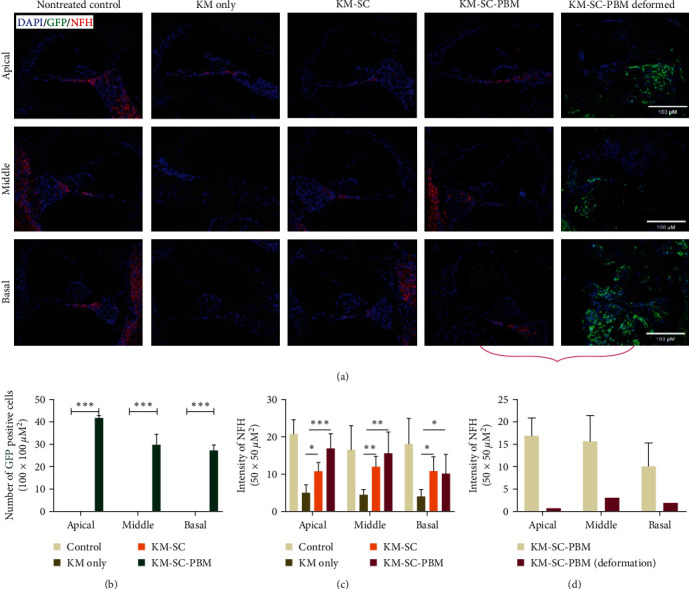
Changes in the peripheral auditory system after combination therapy with mESCs and PBM in a secondary neurodegeneration model. (a) Expression of GFP-mESCs at 2 weeks after combination therapy. NF expression (red) had decreased in the KM only group and increased in the KM-SC and KM-SC-PBM groups. GFP expression (green) in cochlea was observed only in the KM-SC-PBM. One KM-SC-PBM showed abnormal GFP expression in all areas of the cochlea. (b) Quantitative analysis of GFP-positive cells at 2 weeks after combination therapy, only the deformed KM-SC-PBM showed the GFP-positive cell in all turns. (c) Quantitative analysis of NF after combination therapy. The KM-SC-PBM group, except for the deformed KM-SC-PBM sample, showed a higher intensity of NF compared to KM. (d) Comparison of NF between KM-SC-PBM groups and the deformed KM-SC-PBM.  ^*∗*^*p* < 0.05,  ^*∗*^ ^*∗*^*p* < 0.01, and  ^*∗*^ ^*∗*^ ^*∗*^*p* < 0.001. Scale bar = 100 *µ*M. (NF = neurofilament, NFH = neurofilament heavy).

In the peripheral auditory systems in the secondary neurodegeneration model, the survival of cells in the transplanted cochlea showed a lower rate of mESCs in KM-SC than in the KM-SC-PBM group. NF expression had decreased in the KM only group but was found to increase in the KM-SC and KM-SC-PBM groups. GFP expression (green) in the cochlea was observed only in the KM-SC-PBM. One KM-SC-PBM sample showed abnormal GFP expression in all areas of the cochlea (Figure 5(a)) and consistently showed GFP-positive cells in all turns compared to the other groups (Figure 5(b)). The KM-SC-PBM group, excluding the deformed sample, had higher neurofilament (NF) intensity compared to KM only (Figure 5(c)). Even when comparing the NF intensity between the KM-SC-PBM group and the deformed KM-SC-PBM sample, the NF expression rate was higher in the undeformed KM-SC-PBM samples (Figure 5(d)). These results suggest that the combination therapy with PBM can induce neural specification in mESCs transplanted into the cochlea.

### 3.4. Characteristics of GFP-Positive Cells that Underwent Aberrant Proliferation Due to Combination Therapy with mESCs and PBM

**Figure 6:**
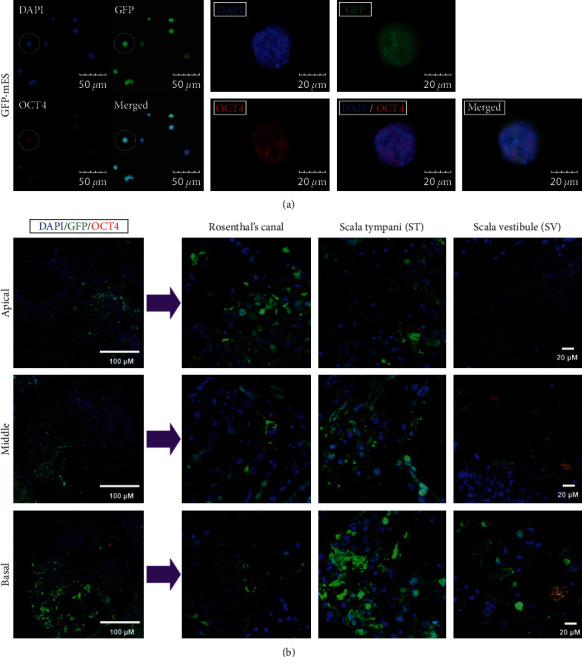
Reduction of pluripotency in extensively proliferated ES cells transplanted into the cochlea following secondary neurodegeneration. (a) Undifferentiated GFP-mESCs used in combination therapy. OCT4 (red color) was expressed in GFP-mESC prior to SC transplantation in vitro. The state of the cells before transplantation is maintained in an undifferentiated state. (b) Characteristics of GFP-mESCs after SC transplantation for the combination therapy. In abnormal GFP expressed KM-SC-PBM, OCT4, which was expressed before SC transplantation into the cochlea, was disappeared in most of GFP expressing cells of cochlea regions including Rosenthal's canal, ST, SV after SC transplantation. Scale bar = (a) 20 *µ*M and (b) 100 and 20 *µ*M.

In this study, undifferentiated GFP-tagged mESCs were used for the combination therapy in the secondary neurodegeneration model. OCT4, which plays an important role in the maintenance of ESC pluripotency, was used to characterize these cells before transplantation (Figure 6). GFP-tagged mESCs expressed OCT4 (red color) maintained this undifferentiated state in vitro prior to transplantation (Figure 6(a)). However, given the aberrant GFP expression in the KM-SC-PBM group, OCT4 expression was no longer detected in most GFP-positive cells in the cochlea (including those in Rosenthal's canal and the ST and SV, Figure 6(b)). These results suggest that the characteristics of mESCs were reduced after transplantation as the cells began to differentiate.

**Figure 7:**
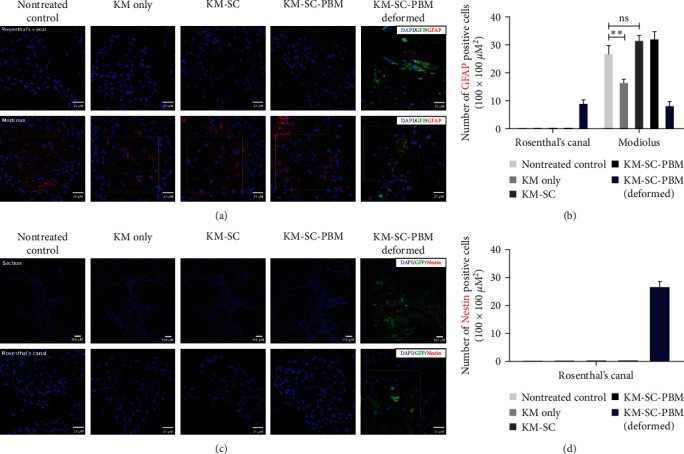
Characterization of the transplanted ESCs in cochlea after 2 weeks of combination treatment. (a) Glial cell expression. Staining of the glial cell marker GFAP was demonstrated after combination therapy. In Rosenthal's canal, only the deformed KM-SC-PBM showed GFAP expression. In the modiolus, GFAP was expressed in all treated groups, and the KM-SC and KM-SC-PBM groups showed increased expression compared to the KM only group, but the deformed KM-SC-PBM group had rather decreased expression. (b) Quantitative analysis of GFAP-positive cells at 2 weeks after combination therapy. In Rosenthal's canal, only the deformed KM-SC-PBM showed increase in GFAP-positive cells compared to the other groups. However, the deformed KM-SC-PBM modiolus had decreased in the number of GFAP-positive cells. (c) Neuronal precursor cell expression. Staining of the neuronal precursor marker Nestin was demonstrated after combination therapy. In both Rosenthal's canal and modiolus, only the deformed KM-SC-PBM showed Nestin expression. (d) Quantitative analysis showed only the deformed KM-SC-PBM showed increase in Nestin-positive cells compared to other groups.  ^*∗*^ ^*∗*^*p* < 0.01. Scale bar = (a) 20 *µ*M and (c) 100 *µ*M.

Neural differentiation was also investigated through additional experiments with GFAP and Nestin expression. Staining of the glial cell marker GFAP was demonstrated after combination therapy. In Rosenthal's canal, only the deformed KM-SC-PBM showed GFAP expression. In the modiolus, GFAP was expressed in all treated groups, and the KM-SC and KM-SC-PBM groups showed an increased expression compared to the KM only group, but the deformed KM-SC-PBM sample had decreased the GFAP expression (Figure 7(a)). Quantitative analysis of GFAP-positive cells at 2 weeks after combination therapy showed only the cells in Rosenthal's canal of the deformed KM-SC-PBM sample showed increase in GFAP-positive cells compared to other groups. However, cells in the modiolus of the deformed KM-SC-PBM sample had decreased GFAP-positive cells (Figure 7(b)).

Staining of the neuronal precursor marker Nestin also demonstrated an increased expression after combination therapy in both Rosenthal's canal and modiolus of the deformed KM-SC-PBM sample (Figure 7(d)). These results suggest that the characteristics of mESCs may alter the neural specification after transplantation.

**Figure 8:**
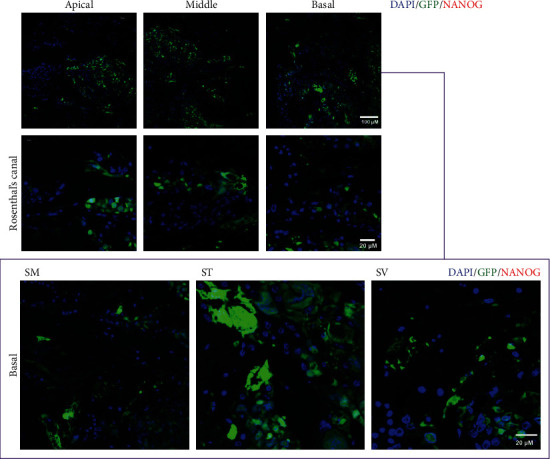
Mouse embryonic stem cells (mESCs) transplanted into the cochlea in the secondary neurodegeneration model proliferated extensively but lacked differentiation capacity and malignancy. Stemness of the transplanted green fluorescent protein (GFP)-tagged mESCs following combination therapy was investigated by the expression of NANOG, a marker of ESC pluripotency or self-renewal function and malignancy in the KM-SC-PBM group. The absence of NANOG expression in transplanted GFP-positive cells in the cochlea after combination therapy suggested that mESC pluripotency was lost after this treatment. Scale bar = 20 *µ*M.

**Figure 9:**
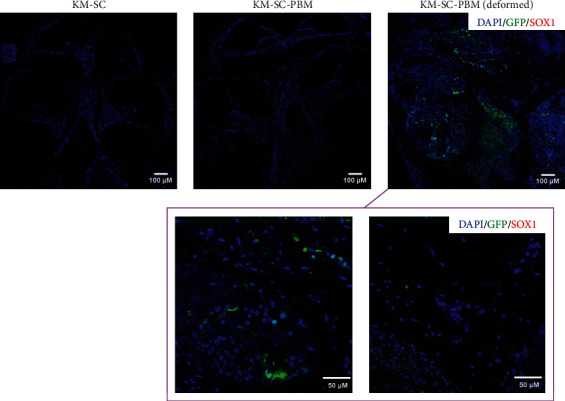
Lack of teratoma formation in extensively proliferated ES cells transplanted into the cochlea after secondary neurodegeneration. Teratoma formation of survived GFP-mESCs in the deformed cochlea after 2 weeks of combination therapy. SOX1, a marker for neuroectoderm formation, was investigated in SC-treated cochleae. SOX1 was not expressed in tested groups including the survived GFP-positive cells of the cochlea. It can be suggested the mESCs did not promote teratoma formation. Scale bar = 50 *µ*M.

The possibility of malignant transformation of the transplanted cells present in the cochlea after combination therapy was also investigated. We examined the expression of NANOG, a marker reflecting ESC pluripotency, or self-renewal function and of malignant cells (indicating tumorigenesis) in the KM-SC-PBM group. No NANOG expression was detected in the transplanted GFP-positive cells in the cochlea after combination therapy (Figure 8). Thus, despite their abundant proliferation, the transplanted mESCs showed no characteristic of malignancy. To determine whether tumors were formed among the transplanted cells after combination therapy, teratoma formation in deformed cochlea was also investigated. A marker for neuroectoderm formation, SOX1, was investigated in SC-treated cochleae. SOX1 was not expressed in all test group including the survived GFP-positive cells of the cochlea. It can be suggested the mESCs did not promote teratoma formation (Figure 9).

## 4. Discussion

Cochlear implantation is used for hearing rehabilitation following sensorineural hearing loss, which is difficult to treat, which involves the transmission of electrical signals via mechanisms other than hair cells [10, 11]. External sound is converted into electrical signals by hair cells and transmitted by spiral ganglion neurons (SGNs) in Rosenthal's canal to the cochlear nucleus of the central nervous system via the modulus nerve bundle. A sufficient number of auditory nerve cells such as SGNs is a very important factor in the efficiency of sound transmission. Therefore, this study focused on changes within Rosenthal's canal due to damage to auditory nerve cells. Because, the prognosis following cochlear implant is very poor when insufficient numbers of auditory nerve cells remained in Rosenthal's canal [12, 13]. Thus, we aimed to improve the connection of cochlear implants to the inner ear by investigating the effects of a combined mESC and PBM treatment to the auditory function and auditory nerve cells in an animal model of secondary neuropathy.

The combined treatment did not improve hearing or increase the auditory nerve response. There is also a possibility that the combined treatment may have damaged hearing. Nevertheless, abundant SC proliferation was observed in animals irradiated via PBM; in other groups, most SCs disappeared immediately after transplantation or were distributed throughout cochlear tissues for short periods of time only.

Here, the neural specificity of ESCs was investigated after 2 weeks of stem cell transplantation and PBM. Some papers have already published that it takes 2 weeks for SC to differentiate into neurons in mice [36], our study conducted experiments under that assumption and found observations related to the effects of the transplanted SC into the inner ear. However, we plan to conduct a longer investigation to concretely confirm the effects of the combined therapy in the recovery of hearing in secondary neuropathy animal model.

The greatest limitation of cochlear SC transplantation is the failure of the cells to persist long enough to differentiate [20, 21]. When SC viability is increased by enhancing the cells' proliferative capacity, the potential problem of cancer development locally or in other parts of the body arises [28–30]. Increasing the number of cells delivered into the cochlea also increases the chances of clogging of the syringe as well as cell agglomeration within the cochlea; thus, it is not recommended. In general, for cell therapy, an adequate cell count is required and the cells are usually in solution. Since the amount of cochlear perilymph fluid is very small only 0.6 *μ*L [37, 38], very few cells can be injected. In the explant coculture study, the number of mESCs used was increased from 60,000 in vivo to 10,000,000 cells per explant in order to compensate the higher volume of media needed for a 35-mm culture plate. Nevertheless, only a few GFP-positive cells were observed to integrate with the explant after 3 days of coculture. Most of the cells were attached directly to the plate or failed to attach at all, washed out or even deteriorated. Further techniques that can improve cell adhesion are necessary.

After delivery of mESCs, the cells not only survived for a long time in the cochlea but were also distributed evenly throughout the region and integrated with the other cells. This result was confirmed in vitro and in vivo. However, these cells did not restore hearing or differentiate into hair cells, the most important cells for hearing. The examination of NANOG expression by epifluorescence confirmed that the cells did not undergo malignant mutation. However, the delivery of mESCs resulted in a deformed cochlea in one of the samples.

The second limitation of SC-based hearing therapy is the difficulty of achieving SC differentiation [39]. In this study, treated cochlea retained GFP-positive SCs, but factors such as Oct4 expression were not observed. The transplanted SCs appeared to differentiate into other cells, but not the desired auditory tissue. The methods used in this study could not resolve the type of cell that the SCs differentiated into.

Physiological activity in the body is maintained by the interaction of various cells. Undifferentiated mESCs are capable of self-renewal and can differentiate into various cells of the organ of Corti. Up until now, only two studies have shown a functional recovery of the auditory nerve, one utilizing human ESCs and another using an immortal mouse otic neuroblast cell [40]. The cell therapy effect in this study was intended using undifferentiated mESCs; however, the transplantation led to the formation of abnormal structures in the cochlea. Neural progenitor cells (NPCs) could be a better alternative for such a SC therapy as these are progenitor cells in the nervous system that include glial cells and neurons but not non-neuronal cells. It is very important to elucidate the molecular mechanisms during organ development in order to improve the efficiency and safety of stem cell therapy. Meanwhile, other groups reported that PBM induced the differentiation and proliferation of bone/cartilage tissue [41–43], but the parameters of PBM differ across the studies.

The cochlea is also composed of bone tissue; thus, PBM may ultimately induce the required SC differentiation and proliferation in this tissue causing the malformations observed in one of the KM-SC-PBM sample. The possibility that various toxic substances used in this study damaged the bone tissue can also be considered; the SCs were applied in the process of repairing this damage, and PBM altered the whole process. Overall, PBM shows great potential for the induction of SC differentiation and proliferation, and if its administration is adjusted appropriately, it may achieve the desired nerve regeneration. Future work will focus on auditory nerve regeneration using specific substances such as nerve growth factors.

## 5. Conclusion

In this study, coculture of cochlear explants and mESCs were confirmed to be connected or in contact, and axon-like structures were identified in some cases, after. A combination treatment with mESCs and PBM in an animal model of secondary neuropathy resulted in the maintenance and integration of SCs into the cochlea in vivo, but no restoration of auditory function.

In addition, PBM appears to have induced the explosive proliferation of SCs transplanted into the cochlea. These cells differentiated into noncarcinogenic cells other than auditory cells. Thus, the results of this study suggest that PBM-related regulation can improve the proliferative capacity of SCs.

## Data Availability

The data that support the findings of this study are available upon request from the corresponding author.

## Supplementary Materials

Supplementary Materials Figure S1: comparison of viable cell of OC between KM only and KM-SC-PBM after combination treatment.
