# Supplementary material for: Photobiomodulation Can Enhance Stem Cell Viability in Cochlea with Auditory Neuropathy but Does Not Restore Hearing
Source: Stem Cells Int. 2023 Nov 15;2023:6845571. doi: 10.1155/2023/6845571 (PMC10665102; doi:10.1155/2023/6845571)
Supplement: Supplementary Materials — Figure S1: comparison of viable cell of OC between KM only and KM-SC-PBM after combination treatment. [file 6845571.f1.docx]

**Supplementary Figure 1. Comparison of viable cell of OC between KM only and KM-SC-PBM after combination treatment.**


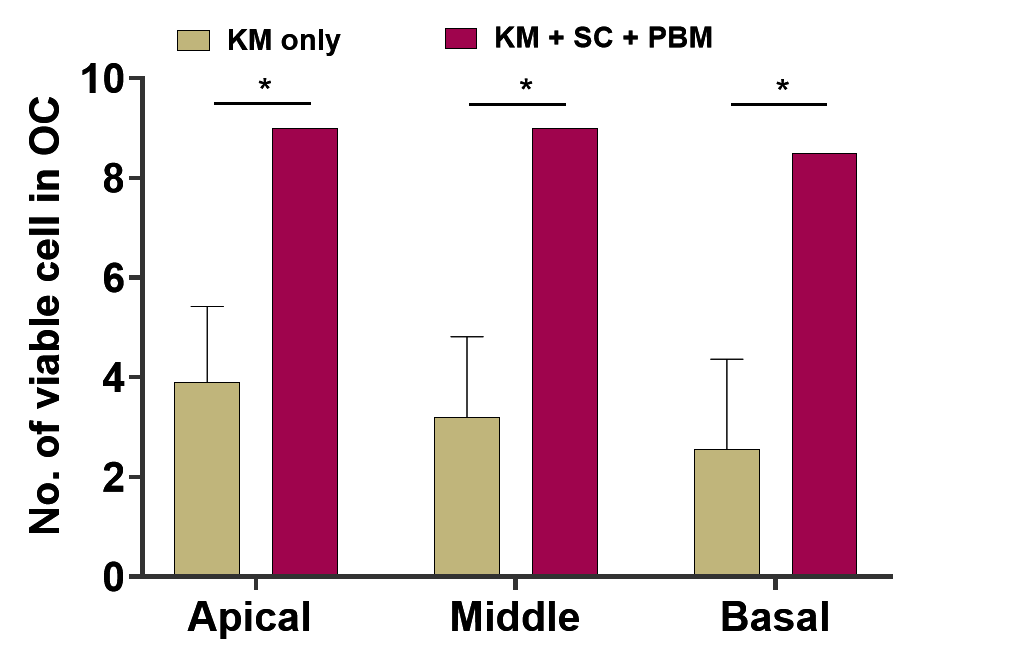


*p < 0.05
